# Human Pulmonary Tuberculosis: Understanding the Immune Response in the Bronchoalveolar System

**DOI:** 10.3390/biom12081148

**Published:** 2022-08-20

**Authors:** María Teresa Herrera, Silvia Guzmán-Beltrán, Karen Bobadilla, Teresa Santos-Mendoza, Mario Alberto Flores-Valdez, Luis Horacio Gutiérrez-González, Yolanda González

**Affiliations:** 1 Department of Microbiology, National Institute for Respiratory Diseases Ismael Cosío Villegas, Mexico City 14080, Mexico; 2 Laboratory of Transcriptomics and Molecular Immunology, National Institute for Respiratory Diseases Ismael Cosío Villegas, Mexico City 14080, Mexico; 3 Biotecnología Médica y Farmacéutica, Centro de Investigación y Asistencia en Tecnología y Diseño del Estado de Jalisco, A.C., Guadalajara 44270, Mexico

**Keywords:** *M. tuberculosis*, bronchoalveolar lavage fluid (BALF), innate and adaptive immune response, active TB, tissue-resident memory T cell, vaccine target

## Abstract

*Mycobacterium tuberculosis*, the causal agent of one of the most devastating infectious diseases worldwide, can evade or modulate the host immune response and remain dormant for many years. In this review, we focus on identifying the local immune response induced in vivo by *M. tuberculosis* in the lungs of patients with active tuberculosis by analyzing data from untouched cells from bronchoalveolar lavage fluid (BALF) or exhaled breath condensate (EBC) samples. The most abundant resident cells in patients with active tuberculosis are macrophages and lymphocytes, which facilitate the recruitment of neutrophils. The cellular response is characterized by an inflammatory state and oxidative stress produced mainly by macrophages and T lymphocytes. In the alveolar microenvironment, the levels of cytokines such as interleukins (IL), chemokines, and matrix metalloproteinases (MMP) are increased compared with healthy patients. The production of cytokines such as interferon (IFN)-γ and IL-17 and specific immunoglobulin (Ig) A and G against *M. tuberculosis* indicate that the adaptive immune response is induced despite the presence of a chronic infection. The role of epithelial cells, the processing and presentation of antigens by macrophages and dendritic cells, as well as the role of tissue-resident memory T cells (Trm) for in situ vaccination remains to be understood.

## 1. Introduction

Pulmonary tuberculosis (TB) is a chronic, infectious-contagious, and difficult-to-control disease caused by *Mycobacterium tuberculosis* (*M. tuberculosis*). More than 100 years after the discovery of *M. tuberculosis* and in spite of the availability of effective treatments for TB, this disease is far from being eradicated. The challenges are diverse and include global TB diagnosis and treatment coverage, effective treatment for TB caused by *M. tuberculosis* strains with resistance to first- and second-line antibiotics, finding markers and/or biomarkers for treatment monitoring, effective vaccines, as well as diseases that compromise the host immune response and have made TB a re-emerging disease. Understanding the local immune response mechanisms involved in the elimination of *M. tuberculosis* will allow for the design of new therapeutic strategies for *M. tuberculosis* [1,2]. Most of what is currently known about the local immune response to TB infection has been derived from animal studies, mainly in mice, or from assays of lung cell lines or peripheral blood cells. Many mechanisms induced during *M. tuberculosis* infection have been identified from studies on bronchoalveolar lavage fluid (BALF) samples.

The lung, as a route of entry for microorganisms, has mechanisms to distinguish pathogens from non-pathogenic organisms. While tolerance is enabled for non-pathogenic organisms, an efficient immune response must be induced to control pathogenic microorganisms and prevent progression to disease. Despite the availability of effective treatments, *M. tuberculosis* has persisted among humans, causing chronic disease. The cells and microenvironment of the lung play an important role in the control of these infections. Understanding the soluble molecules and resident cells present in the lung and how they are modified during respiratory infections, specifically towards *M. tuberculosis* control, will allow for the implementation of interventions for treatment and prevention. The fluid collected during bronchoalveolar lavage allows for the collection of cells, soluble molecules, and volatile and non-volatile compounds induced and/or modified in the lung during infection with *M. tuberculosis* and provides information on the innate and adaptive immune response as well as the characteristics of the alveolar microenvironment. Therefore, in this review, we focus on the mechanisms identified from BALF of TB patients to obtain more accurate information on the innate and adaptive immune responses induced in the alveolar compartment during active TB to identify the cell phenotypes, microenvironment, and differentiation of lung cells.

## 2. *M. tuberculosis* Complex and Its Influence on the Immune Response

Tuberculosis in humans is caused by the members of the *Mycobacterium tuberculosis* complex (MTBC). MTBC is divided into two groups of lineages known as *Mycobacterium tuberculosis* sensu stricto and *Mycobacterium africanum* [3]. Lineages 1–4 of human MTBC correspond to *M. tuberculosis* sensu stricto, and lineages 5 and 6 to *M. africanum*. The more ‘ancient’ evolutionary branches are *M. africanum* and lineage 1. Lineages 2 and 4, which diverged at a later point from a common ancestor and are the modern lineages, are more widely spread compared with the ‘ancient’ lineages [4]. MTBC strains display different cellular and clinical phenotypes and are metabolically diverse and present distinctive levels of virulence and epidemiological consequences [5]. For example, lineages 1 and 3 produce exclusively extrapulmonary tuberculosis disease and lineage 2 is associated to faster development of drug resistance, whereas progress of *M. africanum* infection to active disease is slower than for ‘modern’ lineages. The lineages have an effect on modulation of the inflammatory response; it has been reported that the ‘modern’ lineages (Euro-American, East Asian/Beijing, and Indian/East African) induce lower inflammatory response than those from ‘ancient’ lineages (Indo-Oceanic and West African) [6]. Furthermore, each lineage and specific strain may also influence the course of disease; it has been reported that modern lineages are capable of modulating the IL-1β-induced autophagy [3,7]. Additionally, host’s components also affect the outcome of granuloma, including the spatiotemporal and cellular compositions, as it was recently shown in two non-human primate models [8]. Thus, controversial data on the local immune response could be influenced by lineage, so that correlating the pulmonary immune response with the lineage of the *M. tuberculosis* complex would help to understand the differences in the immune response during pulmonary tuberculosis.

## 3. Alveolar Epithelial Cells (AECs) during *M. tuberculosis* Infection

The route of entry of *M. tuberculosis* into the host is through the respiratory tract to the lung, where the bacteria induce different pathways that modify the immune response and, therefore, the pulmonary microenvironment. Alveolar epithelial cells (AECs) are the first to encounter microorganisms or particles and contribute to the body’s early defenses. AECs provide a physical and immunological barrier and stimulate the acquired immune response [9].

The mechanical clearance of mucus is widely considered the primary innate airway defense, and AECs line the airway surface to facilitate mucus transport through ciliary activity and the regulation of salt and water via transepithelial ion transport [10,11]. Mucus secreted by goblet cells and mucous glands entraps microorganisms or particles propelled by ciliary movement and provides the microenvironment necessary for the activity of antimicrobial substances [12,13,14]. Human AECs participate in innate immunity through the production of reactive oxygen species (ROS) and the secretion of antimicrobial peptides (AMPs) such as α-defensins (human neutrophil peptides (HNP)-1, -4), β-defensins (human beta-defensin (hBD)-1, -2), cathelicidin (antimicrobial peptide LL-37/18-kDa human cathelicidin, LL-37/hCAP18), and chemokine (C-C motif) ligand 20 (CCL20) into the airway surface fluid, with activity against different pathogens including *M. tuberculosis* [9,15].

AECs and alveolar macrophages (AM) are the main cell types in the alveolar space which are in direct contact with inhaled pathogens. There is strong evidence of the ability of *M. tuberculosis* to infect AECs. For instance, in vitro assays have shown that AECs are invaded by *M. tuberculosis*; additionally, *M. tuberculosis* DNA has been found in endothelial cells and AECs in necropsy specimens from lung subjects who died from causes other than TB, suggesting that these cells are a reservoir for *M. tuberculosis* during latent tuberculosis [16]. The evidence suggests that *M. tuberculosis* acquires increased invasiveness for other non-phagocytic cells such as AECs, as it has been reported that *M. tuberculosis*-uptake by A549 cells is less efficient for intracellular control versus professional phagocytes, and *M. tuberculosis* replicates are ~15–20-fold more abundant in AECs than AMs in the same period of time [16]. Although AECs have been identified in BALF samples by microscopic analysis [17], their proportions are low, and their role in the control or pathogenicity of *M. tuberculosis* is not yet fully understood. However, it is known that *M. tuberculosis* enters the AECs and can remain there even in subjects without clinical symptoms; thus, we hypothesize that *M. tuberculosis* may enter the AECs and persist in a latent state. Lung tissue samples from postmortem subjects would provide information to elucidate the role of AECs in active or latent TB. The difficulty in assessing the response of AECs in the human lung during active TB limits our understanding of their role in disease control or as a potential therapeutic target. Consequently, most of our knowledge about pathogenicity is derived from cells that are found in greater proportions, such as macrophages, dendritic cells (DCs), or T cells.

## 4. Cellular and Molecular Composition of the Bronchoalveolar Space of Patients with TB

The microenvironment in the lung is constantly being modified by an influx of molecules including solid particles, microorganisms, and products derived from microorganisms. The induced immune response will depend on the type of microorganism that enters and is mostly non-inflammatory.

**BALF surfactant**. The surfactant coat of the alveolar walls can be recovered from BALF. It is synthesized by alveolar epithelial type 2 (AE2) cells and released following appropriate stimuli by exocytosis from special intracellular storage organelles termed lamellar bodies. The surfactant is composed of lipids (80–90% phospholipids) and proteins (10%). The surfactant proteins (SPs) have been termed SP-A, -B, -C, and -D [18]. AE2 cells also contribute to lung defense by secreting antimicrobial products such as complement, lysozyme, and surfactant proteins [19]. BALF from the involved lobes of patients with TB contain lower levels of SP-A compared to BALF from uninvolved lobes or healthy subjects; a reduction in SP-A levels in pulmonary TB patients has also been reported, and SP-A levels return to normal after TB therapy. Accordingly, it is possible that SP-A reduction is used by *M. tuberculosis* as an evasion mechanism, due to SP-A coating pathogens and inducing agglutination [20].

**Cellular composition of BALF from patients with TB**. The cellular composition of BALF from patients with TB in normal/healthy adult nonsmokers, patients with latent TB, children with active TB, and adults with active TB is shown in Table 1. In active TB disease, both children and adults have a lower percentage of macrophages, while lymphocytes are increased. It is possible that macrophage percentages are not reduced, but that the macrophage–lymphocyte ratio is modified during active disease.

**Figure 1:**
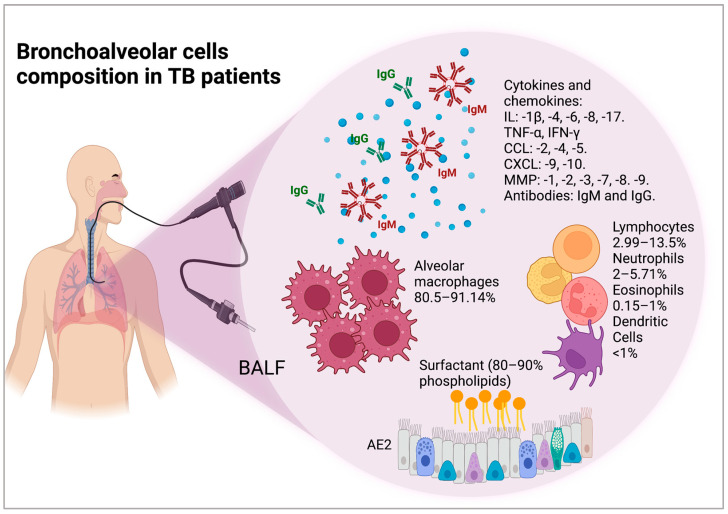
A complex composition of cells of the innate and adaptive immune system is found in bronchoalveolar lavage fluid (BALF) obtained from TB patients, 90% of which comprises alveolar macrophages. Other components found in BALF include surfactants, cytokines, chemokines, and metalloproteinases (created with BioRender.com).

**Soluble molecules present in BALF from patients with TB**. Cytokines such as IL-8, CCL2, and regulated on activation, normal T cell expressed and secreted (RANTES) are present in higher concentrations in BALF from the acute phase of pulmonary TB compared with that of healthy subjects [29]. Additionally, high levels of proinflammatory cytokines, such as IL-1β, IL-6, and tumor necrosis factor (TNF)-α [30], and effector cytokines, such as IFN-γ, IL-4, and IL-17 were identified in the BALF of patients with TB, whereas the Th1 cytokines IFN-γ and TNF-α were present at higher levels in patients with TB compared to healthy subjects but did not differ from other diseases such as sarcoidosis [31,32]. In contrast, another study did not detect IL-17, while higher levels of IL-22 were found in BALF from patients with TB compared with healthy controls [33]. Another study also found elevated IL-1β, IL-4, IL-6, and TNF-α cytokine concentrations in TB patients without differences based on human immunodeficiency virus (HIV) status. However, their clinical status did show differences in BALF: IL-4 and CCL4 levels were higher in HIV-negative patients with moderate-to-severe TB compared to mild TB [34]. Other chemokines found with elevated levels in BALF from active pulmonary TB patients compared to healthy subjects include CCL4 (macrophage inflammatory protein, MIP-1β), chemokine (C-X-C) motif (CXCL)-9 (or monokine induced by gamma interferon, MIG), and CXCL10 (interferon gamma-induced protein 10, IP-10) [34]. The cellular composition of BALF modifies the cytokine levels: low levels of polymorphonuclear neutrophil (PMN) cells are associated with higher IP-10 concentrations [35]. Clinical complications such as acute respiratory distress syndrome (ARDS) also modify the lung microenvironment; it was observed that CXCL8 levels in BALF were significantly higher in an ARDS + TB group compared to TB and ARDS alone groups, although CXCL8 was also increased in both pathologies [36]. It has also been found that MMPs, such as MMP-1, are increased in BALF and correlated with extensive lung damage, and that neutrophil-derived MMP-8 and -9 are increased in TB patients and associated with cavitary disease [37]. Additionally, MMP-1, -2, -3, -7, -8, and -9 concentrations in BALF are increased in patients with TB compared to respiratory control subjects with a diverse range of pulmonary diagnoses, including smoking-related bronchitis, pneumonia, carcinoma, sarcoidosis, foreign body aspiration, and pulmonary vasculitis [38] (Figure 1).

***M. tuberculosis*-specific antibodies in BALF**. It has been reported that while B cell and antibody deficiencies are not risk factors for human TB, mice lacking B cells or the ability to secrete antibodies are more susceptible to infection. Antibodies are involved in other immunological mechanisms such as several enhanced macrophage responses against intracellular *M. tuberculosis*, including phagolysosomal maturation and inflammasome activation independent of pyroptosis [39]. In BALF, higher levels of bacillus Calmette–Guérin (BCG)-specific IgG antibodies from healthy subjects and patients with TB history under a BCG lung challenged in a model of vaccination have been reported [40]. Additionally, IgA and IgG for 38-Da+ lipoarabinomannan (LAM) were higher in the TB than in the non-TB group [41], and *M. tuberculosis*-specific IgA and IgG are increased in BALF [42] (Figure 1).

## 5. Antioxidant Status of the Bronchoalveolar Space of Patients with TB

The phagocytosis of *M. tuberculosis* exacerbates the production of ROS and reactive nitrogen species (RNS) in the host cells by activating mononuclear and polymorphonuclear phagocytes with the respiratory burst mechanism [43,44].

Exacerbated levels of ROS and RNS in pulmonary TB induce a reduction in antioxidant effectors [45], generating an imbalance in oxidant/antioxidant conditions that can cause injury to host tissue and provoke both an inflammatory state and immune suppression that contribute to the development of lung dysfunction [46,47]. Diverse studies report that patients with TB presenting with lipid peroxidation by free radicals may have reduced serum lipid levels [48]. Evidence suggests that an altered lipid profile and low cholesterol level are significantly associated with TB, whereas high cholesterol confers protection against *M. tuberculosis* infection [48,49]. The inflammatory and redox status of patients with pulmonary TB has been extensively studied in plasma or serum; however, systemic parameters hardly represent the lung status.

Given the nature of ROS, which easily react with other molecules in cells, the antioxidant condition is an indirect measure of the oxidative status induced by TB infection. Due to the dilution of the soluble molecules, BALF is not the best sample to identify the oxidative status in TB patients; alternatively, the non-invasive method of sampling airway-lining fluid from exhaled breath condensate (EBC) provides more information since it contains water vapors and hundreds of different compounds of exo- and endogenous origin in trace concentrations [50]. EBC can be transformed into a liquid phase that contains volatile and non-volatile compounds within the aqueous phase. EBC analysis is a unique technique among lung function tests in terms of identifying molecular pathways that reflect airway epithelial function [51]. This sampling technique only requires tidal breathing and is currently accepted as an accessible tool for detecting markers of lung inflammation and oxidative stress [51,52].

**Figure 2:**
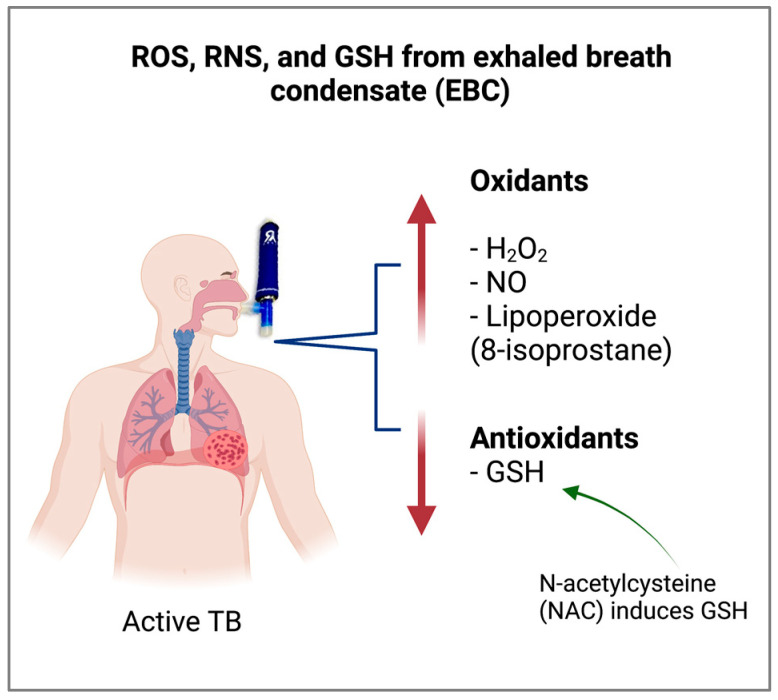
Exhaled breath condensate (EBC) from airway-lining fluid contains water vapors and compounds in trace concentrations. The EBCs from patients with active TB showed increased levels of oxidative stress markers such as reactive oxygen species (ROS), reactive nitrogen species (RNS), and 8-isoprostane, and decreased levels of glutathione (GSH) (created with BioRender.com).

Recently, EBCs of pulmonary TB patients were demonstrated to present significant oxidative stress. The EBCs showed a lower level of glutathione (GSH), a powerful antioxidant that maintains and regulates the thiol-redox status. Furthermore, the EBCs displayed a high level of 8-isoprostane, a prostaglandin that increases the oxidative condition [53] (Figure 2). 8-isoprostane is a reliable biomarker of lipoperoxidation, antioxidant deficiency, and oxidative stress [54]. These parameters reflect the altered oxidizing microenvironment in the airways of patients with TB, contributing to progressive lung disease [55]. The level of hydrogen peroxide (H_2_O_2_) in EBC was significantly higher in TB patients compared to healthy subjects. Furthermore, the level of H_2_O_2_ exhalation significantly decreased in TB patients 2 months after antibiotic treatment. H_2_O_2_ is distinctly produced by alveolar macrophages as well as type 2 pneumocytes in the lower respiratory tract [56]. Nitric oxide (NO) is mainly generated in the bronchial system [56,57]. Patients with active TB exhibited an elevation of exhaled NO with nitric oxide synthase (NOS) induction in alveolar macrophages, and both parameters showed significant diminution following anti-TB treatment [44,58]. ROS and RNS are critical host defense mechanisms for elimination of bacteria during infection [59]. These reactive molecules react with phagosomes and eliminate intracellular pathogens. However, exacerbated ROS and RNS production may cause cellular apoptosis, leading to severe immunopathological outcomes [60].

The EBC of TB patients also showed a high level of inflammatory markers such as nucleosomes, proinflammatory cytokines, and lipid mediators. No correlation was observed between EBC and serum regarding these diverse oxidative and inflammatory markers [53], emphasizing the significance of evaluating local biomarkers of the lung that could be useful in treating pulmonary TB.

Finally, due to the state of exacerbated oxidative stress in the lung in TB patients, a good therapeutic alternative is the use of antioxidants during illness. Improvement of the condition requires appropriately balanced cellular ROS and RNS levels, which are critical for eliminating intracellular mycobacteria without having an injurious effect in the host. For this reason, supplementation with antioxidants may decrease oxidative stress and prevent exacerbated inflammation and pulmonary dysfunction. Studies have shown that the use of antioxidants reduces oxidative stress during *M. tuberculosis* infection, inhibits bacterial growth, and induces an immune response [61]. Some studies have demonstrated that a potent antioxidant such as N-acetylcysteine (NAC) has diverse benefits in mycobacterial infection. NAC treatment moderately increased blood glutathione levels and the serum antioxidant capacity in guinea pigs infected with *M. tuberculosis*, reduced the bacterial burden in the spleen, and decreased the immunopathological severity in the lungs and spleen of animals [62]. Furthermore, in vitro treatment of TB patient blood cells with NAC reduces secretion of cytokines such as IL-1, IL-6, IL-10, and TNF-α [63]. Additionally, clinical trials have shown that the administration of NAC concurrently with anti-TB treatment protects against chemotherapy-induced liver damage in patients [64]. A recent phase II randomized clinical trial in TB patients showed that patients receiving NAC exhibited a significant increase in GSH levels and total antioxidant status while displaying a substantial reduction in lipid peroxidation compared to the control group. Thus, the use of NAC as adjunct therapy could be appraised to alleviate the TB burden and improve clinical management [65]. However, little information is available on whether there is an improvement in oxidative status in the lung of TB patients. It is necessary to monitor the oxidative status during disease progression and TB treatment in order to administer supplementary therapy with antioxidants and reduce the sequelae of TB, thereby improving the patient’s quality of life.

## 6. Alveolar Macrophages in the Bronchoalveolar Space of Patients with TB

Macrophages are the primary host cells for *M. tuberculosis* infection [66,67]. Based on their tissue of origin and the local environment, macrophages can differentiate into specialized cells that perform different functions, including the phagocytosis of pathogens, debris, and dead cells; antigen presentation in association with major histocompatibility complex (MHC-I and MHC-II) molecules; and the production of different types of cytokines to activate or suppress adaptive immune cells [68,69,70]. Distinct phenotypes of macrophages exist in the lung, and these phagocyte variants have been suggested to play different roles in the pathology or control of *M. tuberculosis* infection [71]. Specific resident AM type 1 (M1) capture the mycobacteria within the alveolar spaces [72]. AMs play a role in granuloma formation, *M. tuberculosis* phagocytosis and intracellular growth control, and the progression and dissemination of disease. The initial interaction between *M. tuberculosis* and AMs is crucial to determine whether infection results in bacterial eradication, containment and asymptomatic infection, or unbridled replication and active disease and dissemination.

Little information is available on the response of AMs during TB. The most relevant information is based on alveolar macrophages from patients with TB or healthy subjects infected ex vivo with *M. tuberculosis*. In vitro assays have shown that alveolar macrophages exposed to *M. tuberculosis* induce apoptosis by a mechanism involving TNF-α and increase the survival of *M. tuberculosis* [73].

**Figure 3:**
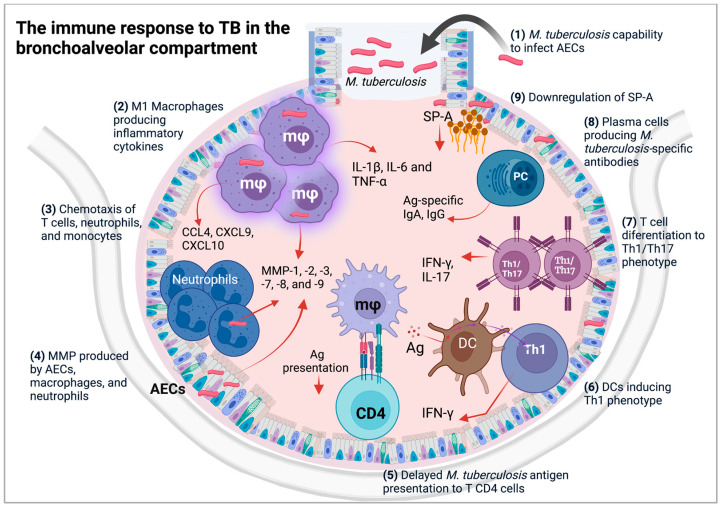
After entering the bronchoalveolar space, *M. tuberculosis* bacilli **(1)** have the ability to infect AECs; **(2)** induce an inflammatory phenotype in macrophages; **(3)** stimulate the production of chemokines for the recruitment of neutrophils, monocytes, and T and B lymphocytes; **(4)** induce the production of metalloproteinases; **(5)** induce a delay in *M. tuberculosis* antigen presentation by macrophages to T lymphocytes; **(6)** induce the differentiation of Th1 lymphocytes through DCs; **(7)** favor the differentiation of Th1/Th17 lymphocytes; **(8)** induce *M. tuberculosis* IgA and IgG antibodies; and **(9)** decrease the production of surfactant (created with BioRender.com).

*M. tuberculosis* can evade the immune response through phagolysosomal inhibition, which occurs through several mechanisms, such as coronin 1 protein (tryptophan aspartate rich coat protein) retention, low acidic intraphagosomal environment, delay in the acquisition of late endosomal markers, and lysosomal enzymes [74,75]. In the 1970s, Armstrong and Hart reported that after virulent *M. tuberculosis* is phagocytosed, it resides in the phagosomes of murine macrophages and blocks phagosome maturation by inhibiting its fusion with lysosomes [76]. This observation was confirmed in alveolar macrophages isolated from patients with active TB, in which lysosomal cargo was not delivered to mycobacterial phagosomes [77,78]. These findings suggest that *M. tuberculosis* is capable of persisting and remaining latent in the host [79], and promote a delay in mycobacterial processing and the formation of peptide–MHC class II complexes, indispensable for antigenic presentation to CD4+ T cells [80,81], which in turn favors *M. tuberculosis* replication and leads to active disease (Figure 3).

Additionally, antimicrobial hydrolases are abundant in alveolar lining fluid (ALF). Exposure of *M. tuberculosis* to ALF can increase phagosome–lysosome fusion and acidification in macrophages; thus, controlling the infection [82,83]. This dual role of phagolysosomal dynamics characterizes the onset of an adaptive immune response with consequent *M. tuberculosis* eradication.

## 7. Alveolar Dendritic Cells (DCs) in the Bronchoalveolar Space of Patients with TB

DCs have an essential role in the defense of the lung based on their anatomical location throughout the respiratory tract as a dense cellular network strategically located for antigen uptake, both within and beneath the epithelial barrier and in the alveoli, which creates a functional cellular interface between the external environment and the internal lung microenvironment [84]. DCs are the most potent antigen-presenting cells with the unique capacity to prime naïve T cells [85]. After encountering antigens in the presence of inflammatory stimuli, peripheral DCs undergo maturation while migrating to the draining lymph node (LN) to present antigens to T cells, initiating adaptive immune responses [86]. During maturation, DCs process antigens and acquire several features, including the upregulation of costimulatory molecules such as CD40, CD80, and CD86, upregulation of MHC-II molecules, cytokine secretion, and upregulation of chemokine receptor CCR7, whose ligands CCL19 and CCL21 are highly expressed in LNs [86,87]. These features guarantee the correct onset of T cell activation by DCs and, hence, the onset of the adaptive immune response.

Among the diverse DC populations, myeloid conventional DCs (DCs) and plasmacytoid DCs (pDCs) are predominant in airway surveillance [88,89,90] (Table 2). The alveolar space is mainly composed of macrophages (90%), although DCs are also present [88,91]. pDCs are highly efficient type-I IFN producers but less efficient antigen-presenting cells compared to DCs. DCs are the main antigen-presenting cells in the alveolar space. In addition, interstitial DCs that emit projections to sample alveolar space can also take up antigens and migrate to the LNs to activate T cells [84]. For years, it was thought that *M. tuberculosis* bacilli only reside in macrophages. Now, it is clear that alveolar DCs can also be infected [92]. As mentioned above, macrophages may harbor *M. tuberculosis* bacilli and favor their multiplication, while DCs appear to limit bacillus multiplication. Differential responses to *M. tuberculosis* infection in macrophages and DCs have been reported, including differential cytokine production and expression of genes related to oxidative stress, vesicle trafficking, and phagosomal acidification [93,94].

In basal conditions, DCs are distributed throughout the airways as well as in the lung parenchyma and in minor quantities in the alveoli [84,88]. The location and quantity of alveolar DCs are inherent to their limited accessibility. Analyses of the BALF of healthy donors have revealed less than 1% of DCs in the total leukocyte population [89,91,95]. Different approaches have been used to study DC functions during TB infection. Human lung DCs express several pattern recognition receptors (PRRs) such as dendritic-cell-specific intercellular adhesion molecule-3-grabbing non-integrin (DC-SIGN), complement receptor 3 (CR3), and mannose receptors capable of recognizing diverse *M. tuberculosis* ligands [96,97]. Notably, DC-SIGN+ DCs bearing mycobacterial antigens have been detected in the LNs of TB patients, suggesting that alveolar DCs that recognize *M. tuberculosis* locally migrate to LNs in vivo during natural TB infection in humans [96,97]. In addition, DCs are also present in human tuberculous lesions (granulomas), suggesting their involvement in the induction of a Th1 response in local lesions; this appears to correlate with the reduced number of DCs in the peripheral blood of TB patients compared with healthy donors [96,98,99] (Figure 3). Furthermore, DCs are able to uptake mycobacterial antigens from apoptotic cells, principally apoptotic vesicles derived from TB-infected macrophages; in turn, these DCs can crossprime CD8+ T lymphocytes in favor of TB control [100,101].

Due to the central role of DCs in eliciting an effective adaptive immune response against TB, approaches to generate DC-based TB vaccines have been proposed [102,103]. The rationale behind these approaches is to deliver *M. tuberculosis* antigens directly to DCs in the lung mucosa or favor *M. tuberculosis* antigen uptake by DCs through chimeric molecules targeting DC surface receptors. Another proposed approach involves DCs transduced with attenuated virus carrying *M. tuberculosis* antigens.

Although it is necessary to address several known limitations in the study of alveolar DCs, distinct approaches have been used to better understand the *M. tuberculosis*–DC relationship. Different studies on the ability to maintain peripheral tolerance as source of IL-2 for T-reg cells [104] to improve DCs migration to the lungs or antigen-presenting function to T-cell response [105] have been performed. The knowledge generated is useful not only to better understand the disease but also to find therapeutic alternatives.

## 8. Alveolar Lymphocytes in the Bronchoalveolar Space of Patients with TB

Protection against TB is associated with the development of a Th1 immune response characterized by the production of IFN-γ, which induces alveolar macrophage activation and promotes mycobacterial clearance [106]. The importance of IFN-γ in the control of mycobacterial infections is evidenced in patients with Mendelian susceptibility to mycobacterial disease, who present with high susceptibility to mycobacterial infection and recurrent infections. Thus, mutations in IL-12/IL-23/IFN-γ affect IFN-γ production by T lymphocytes and, consequently, macrophage activation is reduced and mycobacterial infection progresses to active disease [107,108].

Analysis of bronchoalveolar cells (BACs) in BALF from pulmonary TB patients has revealed the cellular composition in the alveolar compartment, comprising macrophages, lymphocytes, and neutrophils; additionally, lymphocytes and neutrophils are increased in patients compared with healthy controls [109,110,111].

Compartmentalization of the immune response is observed in pulmonary TB patients, since BACs stimulated with mycobacterial antigens such as purified protein derivative (PPD), antigen 85 complex (Ag85), or mannose-capped lipoarabinomannan (ManLAM) show increased proliferation. Moreover, IFN-γ producer cells are present among BACs and alveolar lymphocytes respond to PPD stimulation [110].

BALF from pulmonary TB patients shows an increase in *M. tuberculosis*-specific T lymphocytes that have a memory phenotype (C8+CD45RO+/CD4+CD45RO+), but CD4+CD45RO+ constitutes the main source of IFN-γ in supernatants [109,112]. In TB patients, the percentage of Th1 alveolar lymphocytes (CD4+IFN-γ+) is increased in comparison with non-TB subjects, Th1 cells were identified after phorbol myristate acetate (PMA) plus ionomycin in vitro stimulation [111] (Figure 3). Additionally, lymphocyte populations present in the lung of pulmonary TB patients are Th1 (CD4+IFN-γ+CXCR3−), Th17 (CD4+IL-17A+CXCR3−), and Th1/Th17 (CD4+IFN-γ+IL-17A+CXCR3−). These lymphocytes are PPD-specific producers of IFN-γ, IL-17A, and IFN-γ/IL-17A, with Th1 being the main producer of local IFN-γ [113,114]. Thus, Th1 has an important protective function in TB. However, when T lymphocytes producing IFN-γ or IL17A contribute to an exacerbated immune response, lung tissue damage and promotion of *M. tuberculosis* dissemination may occur. In pulmonary TB patients, the exacerbated inflammatory response can be regulated by alveolar Treg (CD4+CD25+FOXP3+); this population is increased in active TB patients in comparison with latent TB, and both T cell proliferation and mycobacterial growth restriction in infected macrophages are suppressed [115,116].

Another report suggests that BALF from pulmonary TB is characterized by a Th1/Th2 immune response, and proinflammatory and anti-inflammatory cytokines converge in the alveolar site. Cytokines such as IFN-γ are produced mainly by the Th1-phenotype whereas IL-4 is produced by the Th2 phenotype. Although Th1-type cells produce IFN-γ and TNF-α to mediate antituberculous responses in the lung, a Th2-type response has been reported that could be associated to the tissue necrosis phenomenon related to cavitary evolution of TB [117].

In summary, different specific lymphocyte populations are present in human pulmonary TB, involving Th1/Th2/Th17/Treg cells, which produce cytokines such as IL-1β, -6, -12, -17, IFN-γ, and TNF-α that converge with IL-10 and IL-4 and chemokines such as IL-8, CXCL-9, -10, and CCL4 to control *M. tuberculosis* intracellular growth. However, active TB disease control depends both on the host immune response and the virulence of *M. tuberculosis* through evasion mechanisms that exploit and escape from immune cells.

## 9. Tissue-Resident T Cells and Lung Memory Immunity

The resident immune response cells of the lung have particular characteristics that allow them to reside in the lung and respond immediately to the entry of pathogens. The majority of T cells in the human lung are tissue-resident memory T cells (Trms), which act as important long-lived sentinels that protect against invading pathogens and contribute to protection in the context of vaccination [118].

Trms are a population of T cells that have functional and transcriptional similarities to central and effector memory T cells and reside in the lung for prolonged periods, being a first line of defense against subsequent infection in these tissues [119].

**Figure 4:**
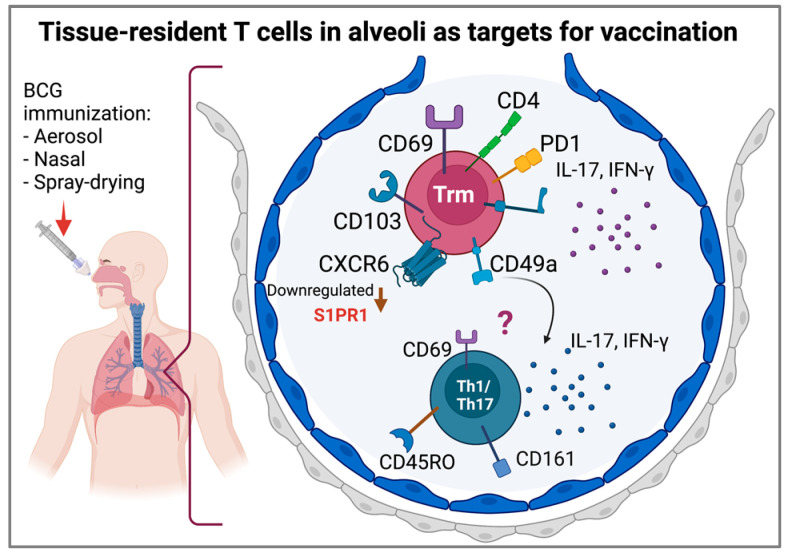
In healthy human lungs, Trms express markers for retention such as CD69, CD103, CD49a, CXCR6, and PD-1 and produce the cytokines IL-17 and IFN-γ. Additionally, a T cell subset with a memory Th1/Th17 phenotype, possibly derived from Trms, has been detected. Both T cell subtypes have been proposed as vaccine targets (created with BioRender.com).

In healthy human lungs, Trms express markers for retention, adhesion, and migration to tissues, such as CD69, CD103, CD49a, and CXCR6, produce IFN-γ and IL-17, and express programmed death receptor (PD-1) and CD101 molecules [120]. Trm cells are derived from precursors that entered tissues during the effector phase of the immune response and remained positioned within this compartment; they express CD69 and CD103 and downregulate the sphingosine-1-phosphate receptor 1 (S1PR1), preventing cells from responding to sphingosine-1-phosphate (SIP) and consequently preventing tissue egress [121]. In the human lung, CD8+ T cells from the airway and BALF have higher CD103 expression compared to CD8+ T cells from the tissue parenchyma, suggesting that CD103 expression may promote localization in airways due to interactions with epithelial cells [121]. Additionally, Trms express CD44, CXCR3, CD11a, and very late antigen-4 (VLA-4) and downregulate killer cell lectin-like receptor subfamily G member 1 (KLRG1) and CD62L [122]. Additionally, in cells from BALF from healthy subjects, a CD3+CD161+CD45RO+ subset of Trm cells with a Th1/Th17 phenotype has been identified, suggesting a contribution to local immune responses in the lung [123] (Figure 4).

There is no direct evidence for the involvement of Trms in TB; however, it is indirectly known that the frequency of Th17 cells in active TB patients is significantly lower than that in healthy subjects, suggesting that Th17 cells may contribute to protection [124]. In tuberculous pleural effusion (TPE), a predominant CD103+CD8+ T cell subset was identified [125]; nevertheless, the role of Trms in BALF from TB patients remains to be fully characterized. Given the difficulty in assessing Trm generation in response to vaccines in humans, several studies in mice have been performed. The generation of immunity to *M. tuberculosis* through the generation of BCG-induced Trm cells has been reported; since the BCG vaccine protects infants but confers poor protection against pulmonary TB in adolescents and adults, assays using an intradermal murine BCG vaccination model have been developed, inducing a population of antigen-specific CD4+ T cells within the lung parenchyma that persists for >12 months post-vaccination [126].

Additionally, using the prime-boost, adjuvant, and viral vectors of *M. tuberculosis* antigens or BCG, it is possible to generate CD4+ and CD8+ lung Trms that improve protection against TB challenge [127]. In humans, airway challenge with the purified protein derivative (PPD) induces CD4+ Trm cells in BALF, highlighting the potential of Trm targeting in vaccines for tuberculosis [118]. Additionally, airway mucosal boosting with the parenteral H56 candidate fusion protein adjuvanted with CAF01 liposomes (H56:CAF01) immunization induces a population of long-lived lung-resident T cells and increases early vaccine T cell responses to pulmonary *M. tuberculosis* challenge in mice [34,112]. Furthermore, other studies have shown the generation of Trms in different mouse BCG vaccination models [128,129,130]. Trm establishment after vaccination increases protection against pathogens such as *M. tuberculosis* [131], and mucosal vaccine administration should substantially boost BCG immunization. Given the importance of Trms in the TB immune response in mice, future studies are necessary to characterize the frequency and function of Trms in BALF samples from humans and consider their role in novel vaccines.

## 10. The Pulmonary Immune Response and the Granuloma

The granuloma is the body’s response to chronic antigenic stimulants. It is formed by macrophages, epithelioid cells, and multinucleated giant cells, and is surrounded by T lymphocytes [132]. TB granulomas have an interplay between the innate and cellular adaptive immunity [133], in which, after phagocytosis of *M. tuberculosis*, an early inflammatory response is initiated, resulting in the recruitment of cells from blood vessels and neighboring tissues and the subsequent formation of granulomas. The granulomatous lesions provide a niche in which *M. tuberculosis* may infect recruited macrophages and persist for long periods of time via a TNF-α-dependent mechanism [132].

The chemokines CCL2/MCP-1, CCL12, and CCL13 induce the recruitment of macrophages at the granuloma site, which in turn engulfs infected debris; the granuloma is then expanded or a new granuloma in a distal tissue is formed. Additionally, neutrophils recruited by macrophage-produced IL-8 contribute to persistent bacilli, scavenge infected cells, and kill mycobacteria through an NADPH-dependent mechanism. However, TNF-α excess and an exacerbated T-cell immunity (Th1-type) cause macrophage necrosis and the release of mycobacteria into the extracellular space. Then, the collagenase MMP-1 is implicated in granuloma cavitation through the degradation of the fibrous extracellular matrix surrounding the granuloma [132]. Upon specific conditions, *M. tuberculosis* may also induce macrophage apoptosis through the early secretory antigenic target 6 kD (ESAT-6) secretion system 1 (ESX-1), a type VII secretion system implicated in *M. tuberculosis* virulence, which leads to bacterial spread [134]. The granuloma cavitation induces mycobacteria release into the airways, where they can be eliminated by cough and infect a new host. All these events contribute to the persistence of *M. tuberculosis* in TB patients [133,134]. Granuloma formation is a mechanism for the control of *M. tuberculosis*; however, the local mechanisms associated with containment of infection or granuloma disorganization that contribute to active disease remain to be determined.

## 11. Local Administration of Live Attenuated Vaccines against Pulmonary TB

BCG is currently applied as an intradermal injection to newborns and infants. Because TB is transmitted via the inhalation of infectious droplets containing *M. tuberculosis*, for some years now, delivery of BCG (or other novel modified rBCG or live attenuated vaccine candidates) via the aerosol, intranasal, or intratracheal routes has been explored as a potential improvement to protect against this disease. In this regard, it is worth noting that in guinea pigs, aerosol delivery of BCG induced a substantially improved protective effect against TB compared with intradermal delivery [135]. On the other hand, intranasal but not subcutaneous administration of BCG conferred robust protection against pulmonary TB challenge in susceptible DBA/2 mice [136].

Recently, it was shown that aerosol immunization of macaques with an *M. tuberculosis* mutant (in the sigH gene; MtbΔsigH) reduced the bacterial burden of *M. tuberculosis* by approximately 3-log10 while significantly diminishing clinical manifestations and granulomatous pathology [137]. Similarly, direct pulmonary mucosal BCG delivery reduced TB disease where standard intradermal injection failed [138], even when rhesus macaques were exposed to a very limited infectious dose in a repeated manner [139].

On a related topic, an aerosol prime followed by an intradermal boost regime with modified vaccinia Ankara 85A (MVA85A) was well tolerated in a phase I randomized controlled clinical trial conducted in the United Kingdom, whereas an intradermal prime followed by an aerosol boost resulted in transient but significant respiratory adverse effects [140]; thus, suggesting that the former scheme has potential for evaluation of efficacy against TB in humans. In fact, inhaled aerosol delivery of human serotype-5 Ad-vectored TB vaccine (AdHu5Ag85A) in humans induced polyfunctional airway tissue-resident memory CD4 and CD8 T cells, whereas intramuscular injection failed to do so [141].

BCG has also been shown to be rendered more attenuated by delipidation so that it can be directly delivered to the lungs, likely reducing harmful inflammation that would be more pronounced in already infected patients [142]. It is worth noting that several devices that would allow aerosol delivery of BCG have recently been tested for their effects on viability, which would most likely impact immunogenicity and protection afforded in subjects receiving vaccines [143]. Additionally, spray-drying to produce a dry powder respiratory delivery method for BCG has recently been reported [144]. The efficacy of protection against TB of this formulation remains to be determined.

Taken together, these studies highlight the potential benefits of aerogenic vaccination with BCG or other regimes. However, despite these encouraging results, it should be noted that in rhesus macaques, no difference was found in the survival of animals vaccinated with BCG via the aerosol or intradermal routes when challenged with an ultralow dose of *M. tuberculosis* Erdman strain (estimated in four colony-forming units (CFU) [145]. These results prompt us to wonder what factor(s) could underlie these differences, although it is true that only one report, as compared to several mentioned here, shows lack of improvement while using an aerogenic route of vaccination.

Nevertheless, we should consider that experiments were conducted in different hosts (guinea pigs, mice, and non-human primates) using different aerogenic delivery routes (intranasal, pulmonary, and aerosol), which may impact the actual dose of vaccine reaching the lungs. Furthermore, additional variations could stem from the challenge dose. All these factors have already been suggested for consideration during preclinical evaluations of TB vaccine candidates [146].

Addressing the local immune response in humans may present several difficulties; among them, we can envision (1) invasiveness or discomfort induced to otherwise healthy, vaccinated people during BALF sample collection; (2) appropriate timing for sampling BALF—as there is no current effective correlate of protection (CoP), any measure of the immune response (innate or adaptive) determined at a single time point after vaccination may or may not be associated with protection against disease later in life; (3) variability of the sub-strain of BCG administered in different countries, as it has recently been shown that the most widely applied sub-strains (Danish, Japanese, and Russian) differ in their viability and capacity to induce ex vivo (peripheral) immune responses [147]—thus, it can be hypothesized that responses in BALF may as well differ depending on what BCG was administered. We do not mean to rule out the evaluation of immune responses in BALF upon vaccination, but rather highlight some of the major points worth considering in evaluating the usefulness of this sample in predicting protection against disease.

## 12. Concluding Remarks

The innate and adaptive immune response induced during *M. tuberculosis* infection is fairly well understood in in vitro and animal models; however, further study of the local immune response in humans in vivo is required to understand the mechanisms of adaptation and evasion of *M. tuberculosis*. Some critical points that require further investigation are:

- The role of AECs in the local response to *M. tuberculosis*.
- The phenotypes of macrophages and DCs and the processing and presentation of mycobacterial antigens during different phases of the disease.
- The immune response associated with the characteristics of patients and their comorbidities.
- The relationship between lineages of the *M. tuberculosis* complex with the lung immune response during active TB.
- Assessing the local immune response at different times after vaccination and exploring the memory immune response induced by BCG sub-strains used for vaccination.

## Figures and Tables

**Table 1:** Cellular composition of BALF from healthy subjects, children, and adult patients with latent and active TB.

Cell Type	Percentage	Condition	Reference
Alveolar macrophages	>85%	Healthy *	[21,22]
Lymphocytes	10–15%	Healthy *	[21,22]
CD4:CD8 ratio	0.9–2.5	Healthy *	[21,22]
Neutrophils	<3%	Healthy *	[21,22]
Eosinophils	<1%	Healthy *	[21,22]
Epithelial cells **	<5%	Healthy *	[21,22]
Alveolar macrophages	80.5–91.14%	Latent TB	[23,24]
Lymphocytes	2.99–13.5%	Latent TB	[23,24]
CD4:CD8 ratio	3.14	Latent TB	[23,24]
Neutrophils	2–5.71%	Latent TB	[23,24]
Eosinophils	0.15–1%	Latent TB	[23,24]
Alveolar macrophages	70.60%	Children with TB ***	[25]
Lymphocytes	20.20%	Children with TB ***	[25]
Neutrophils	9.20%	Children with TB ***	[25]
Alveolar macrophages	52.4–66.7%	Adult with TB	[26,27,28]
Lymphocytes	12.2–33%	Adult with TB	[26,27,28]
Neutrophils	5.30%	Adult with TB	[26,27,28]
Eosinophils	2.90%	Adult with TB	[26,27,28]

* Adult nonsmokers; ** squamous epithelial/ciliated columnar epithelial cells; *** aged 3 months to 13 years.

**Table 2:** Phenotypes of alveolar DCs.

DCs	Phenotype	Location
Myeloid conventional DC1	CD11c+ CD14− MHC-II+ CD1c+	Alveoli/interstitium
Myeloid conventional DC2	CD11c+ CD14− MHC-II+ CD141+	Alveoli/interstitium
Plasmacytoid DC	CD11c− CD14− MHC-II+ CD123+	Interstitium

## Data Availability

All data supporting this review are cited.

## Abbreviations

AdHu5Ag85A, human serotype-5 Ad-vectored TB vaccine; AEC, airway epithelial cell; AE2, alveolar epithelial type 2 cell; Ag85, antigen 85 complex; ALF, alveolar lining fluid; AM, alveolar macrophage; AMP, antimicrobial peptide; ARDS, acute respiratory distress syndrome; BALF, bronchoalveolar lavage fluid; BCG, bacillus Calmette–Guérin; BAC, bronchoalveolar cell; CAF01, cationic adjuvant formulation 01; CCL, chemokines (C-C motif) ligand; CXCL, chemokines (C-X-C motif) ligand; CoP, correlate of protection; CR3, complement receptor 3; DBA/2, Dilute Brown Non-Agouti; DC, dendritic cell; DC-SIGN, dendritic cell-specific intercellular adhesion molecule-3-grabbing non-integrin; EBC, exhaled breath condensate; ESAT-6, early secretory antigenic target 6 kD; ESX-1, ESAT-6 secretion system 1; GSH, glutathione; H_2_O_2_, hydrogen peroxide; H56:CAF01, H56 candidate fusion protein adjuvanted with CAF01 liposomes; hBD, human beta-defensin; hCAP18, 18 kDa human cathelicidin; HIV, human immunodeficiency virus; HNP, human neutrophil peptide; IFN, interferon; Ig, immunoglobulin; IL, interleukin; IP-10, interferon gamma-induced protein 10; KLRG1, killer cell lectin-like receptor subfamily G member 1; LAM, lipoarabinomannan; LL-37, human antimicrobial peptide; LH, lymph node; M1, alveolar macrophage type 1; ManLAM, mannose-capped lipoarabinomannan; MHC, major histocompatibility complex; MIG, monokine induced by gamma interferon; MIP, macrophage inflammatory protein; MMP, matrix metalloproteinase; MVA85A, modified vaccinia Ankara 85A; *M. tuberculosis*, *Mycobacterium tuberculosis*; NAC, N-acetylcysteine; NO, nitric oxide; NOS, nitric oxide synthase; PD-1, programmed death receptor; pDC, plasmacytoid dendritic cell; PMA, phorbol myristate acetate; PMN, polymorphonuclear neutrophil cell; PPD, purified protein derivative; PPR, pattern recognition receptor; RANTES, regulated on activation, normal T cell expressed and secreted; RNS, reactive nitrogen species; ROS, reactive oxygen species; SIP, sphingosine-1-phosphate; S1PR1, sphingosine-1-phosphate receptor 1; SP, surfactant protein; TB, tuberculosis; TNF, tumor necrosis factor; TPE, tuberculous pleural effusion; Trm, tissue-resident memory T cells; VLA-4, very late antigen-4.
